# Determinants of health facility delivery among young mothers in Ghana; insights from the 2014 Ghana Demographic and Health Survey

**DOI:** 10.1186/s12884-022-04985-5

**Published:** 2022-08-20

**Authors:** Emmanuel Anongeba Anaba, Deda Ogum Alangea, Adolphina Addo-Lartey, Emefa Judith Modey, Adom Manu, Stanley Kofi Alor, Kwasi Torpey

**Affiliations:** 1 Department of Population, Family and Reproductive Health, School of Public Health, University of Ghana, Accra, Ghana; 2 Department of Epidemiology and Disease Control, School of Public Health, University of Ghana, Accra, Ghana; 3 Department of Social and Behavioral Sciences, School of Public Health, University of Ghana, Accra, Ghana

**Keywords:** Young mothers, Health facility delivery, Ghana, Demographic and health survey

## Abstract

**Background:**

Globally, young women deliver at home, often under unhygienic conditions and without skilled birth attendants. This study identified the determinants of health facility delivery among young mothers in Ghana.

**Methods:**

We analysed secondary data from the 2014 Ghana Demographic and Health Survey, which collected data across the former ten administrative regions of Ghana. This study analysed data from the ‘women file’ by adjusting for the sample weight. STATA/SE version 16 was employed to analyse the data by computing descriptive statistics, Chi-square, and Binary Logistic Regression.

**Results:**

Seven in ten young mothers gave birth in a health facility. Young mothers who had secondary school education were over three-fold more likely to deliver in a health facility (AOR = 3.5, 95% CI: 1.33–9.23) compared with young mothers with no formal education. Young mothers who resided in rural areas had lower odds (73%) of delivering in a health facility (AOR = 0.27; 95% CI: 0.14–0.514) compared with those in urban areas. Young mothers within the richest wealth quintile also had higher odds (8 times) of delivering in a health facility (AOR = 8.24; 95% CI: 0.95–71.77) compared with those within the poorest wealth quintile. Young mothers who obtained four to seven antenatal visits (AOR = 0.53; 95% CI: 0.27–1.03) had lower odds of delivering in a health facility compared with those who obtained eight or more antenatal visits.

**Conclusion:**

The majority of young mothers in Ghana gave birth in a health facility. The likelihood of delivering in a health facility was influenced by socio-demographic factors, economic factors and utilization of antenatal care services. Therefore, interventions aimed at increasing utilization of skilled delivery among young women should focus on promoting girl child education, economic status and antenatal care visits.

## Introduction

Maternal and newborn deaths are global health problems, but common in sub-Saharan Africa (SSA) [1, 2]. For instance, about two-thirds of global maternal deaths occur in SSA [1–3]. Reducing maternal and newborn mortalities remains a global health priority. For example, the Sustainable Development Goals, especially Target 3.1 seeks to reduce the global Maternal Mortality Rate (MMR) to less than 70 per 100,000 live births by 2030, while Target 3.2 seeks to end preventable deaths of newborns by 2030 [4]. Studies across the globe have shown that younger age is a major risk factor for maternal and newborn deaths [5, 6]. Young mothers (10–24 years) are at a higher risk of severe complications during childbirth, including infections and eclampsia [7]. Globally, pregnancy and childbirth complications are the top causes of mortality among mothers aged 15–19 years [8, 9]. Also, children born to young mothers are at a higher risk of death and severe neonatal conditions, preterm delivery, and being underweight [8, 10].

Health facility delivery or skilled birth attendance has proven to be an effective intervention in reducing maternal and newborn deaths. Health facilities have professional birth attendants (i.e. nurses, doctors, and midwives), infrastructure, logistics, and referral systems to help in the provision of quality health services during labour, delivery, and the postpartum period [11]. In addition, skilled birth attendants are trained to manage obstetric complications [12]. Yet, the utilization of skilled birth attendance among young mothers remains low in low and middle-income countries [13, 14]. For instance, a cross-sectional study in the Northern region of Ghana revealed that 22.1% of young women in rural areas and 36.7% of those in urban areas used skilled delivery services [15]. Factors associated with the utilization of skilled delivery include educational status, health insurance status, optimal antenatal care visits, and socioeconomic status. Other correlates include the place of residence, previous delivery, exposure to mass media, religion, and partners’ educational status [7, 16].

In Ghana, the maternal mortality rate in 2017 was estimated at 309 per 100,000 live births [17]. which is higher than the global target of less than 70 per 100,000 by 2030. In addition, teenage pregnancy remains a serious public health concern. In 2020 alone, 109,888 Ghanaian girls between the ages of 10–19 years were impregnated [18]. However, little is known about their place of delivery. Existing studies on skilled birth attendance focused on all women of reproductive age without age-specific estimates [19, 20]. However, young people have different healthcare needs and health-seeking behaviours, hence limiting the generalizability of findings from the adult population to the young population. The objectives of this study were to assess the prevalence of health facility delivery, and associated factors among mothers aged 15–24 years in Ghana, using national representative data. Findings from this study will help inform maternal health policies and programming, which can help reduce the burden of maternal and neonatal mortalities. We hypothesize that young mothers from poor households and rural areas are less likely to give birth in a health facility compared with their counterparts.

## Methods

### Source of data

This study analysed data from the 2014 Ghana Demographic and Health Survey (GDHS). The survey collected data across the former ten administrative regions of Ghana, using structured questionnaires, from both men and women in rural and urban areas. Women between the ages of 15–49 years were the target population. The study participants were selected using a two-stage sampling procedure. First, 427 clusters were selected from the updated version of the 2010 Population and Housing Census sampling frame. Second, about 30 households were selected from each cluster using a systematic sampling technique proportional to size. In all, 3150 women aged 15–24 years participated in the 2014 GDHS. This present study focused on women aged 15–24 years who had delivered in the last five years to the survey. Therefore, women who fell outside this criterion were excluded from the analysis. A weighted sample of 864 women aged 15–24 years was included in this analysis. The 2014 GDHS obtained written informed consent from the caregivers of minors (15–17 years) and adults. The survey received approval from the Ghana Health Service Ethics Review Committee. Further information about the 2014 GDHS is provided in the full report [21].

### Measures

In this study, the main outcome was the place of delivery (*where did you give birth?*). This variable was recoded as (1 = health facility and 0 = home). The exposure variables found in the literature included socio-demographic factors, insurance coverage, utilization of antenatal care, exposure to mass media (frequency of reading newspaper/magazine, listening to radio, and watching television), and barriers to accessing healthcare. The socio-demographic variables included the following: age, educational status, marital status, wealth index, place of residence, geographical region, religion, employment status, partner’s educational status, health insurance coverage and distance to a health facility.

### Statistical analysis

STATA/SE, version 16 (StataCorp, College Station, Texas, USA) was employed to analyse the data. Before the analysis, some variables (i.e. religion, age, marital study, employment status, and ANC visits) were recorded. The sampling weights, clustering, and stratification (complex survey design) were taken into consideration during the analysis by using the ‘*svy*’ STATA command, which fits statistical models for complex survey data by adjusting the results of a command for survey settings identified through ‘*svyse*t’ [22]. Both descriptive statistics and inferential statistics were computed. At the univariate level, frequency and percentage were computed, while Chi-square analysis was computed at the bivariate level. Adjusted odds ratios were computed at the multivariable level employing binary logistic regression analysis. All statistical significance was reported at the 0.05 significance level.

## Results

### Descriptive statistics

The majority of the participants were aged 20–24 years (79%), married (63%), and resided in rural areas (61%). More than half of the participants professed Christianity (78%). Also, six in ten participants were employed and 62% were covered by health insurance. Exactly, 71% had no distance-related problem in accessing health services. Regarding exposure to mass media, the majority of the participants did not read a newspaper (90%), 19% did not listen to radio and 27% did not watch television. In addition, the majority of the participants obtained between 4 to 7 antenatal visits (60%). Exactly, 75% of the participants delivered in a health facility, while 25% delivered at home (Table 1).

**Table 1:** Socio-demographic characteristics of participants

Characteristic	Frequency	Percentage
**Age (years)**		
15–19	178	21
20–24	686	79
**Education status**		
No education	154	18
Primary	203	24
Senior high	496	57
Higher	11	1
**Marital status**		
Unmarried	320	37
Married	544	63
**Wealth index**		
Poorest	187	22
Poorer	215	25
Middle	221	25
Richer	155	18
Richest	86	10
**Areas of residence**		
Urban	341	39
Rural	523	61
**Region**		
Western	95	11
Central	102	12
Greater Accra	109	13
Volta	74	8
Eastern	100	12
Ashanti	129	15
Brong-Ahafo	97	11
Northern	97	11
Upper East	38	4
Upper West	23	3
**Religion**		
Christianity	669	78
Islam	133	15
Traditional	21	2
No religion	41	5
**Employment status**		
Unemployed	302	35
Employed	562	65
**Covered by health insurance**		
No	326	38
Yes	538	62
**Husband/partner education**		
No education	130	22
Primary	75	12
Senior high	379	62
Higher	26	4
**Distance to a health facility**		
Big problem	250	29
Not a big problem	614	71
**Frequency of reading newspaper**		
Not at all	774	90
Less than once a week	54	6
At least once a week	35	4
**Frequency of listening to the radio**		
Not at all	161	19
Less than once a week	301	35
At least once a week	402	46
**Frequency of watching television**		
Not at all	233	27
Less than once a week	234	27
At least once a week	397	46
**Antenatal care (ANC)**		
No visit	24	3
1–3 visits	121	14
4–7 visits	520	60
8–20 visits	199	23
**Place of delivery**		
Health facility	645	75
Home	219	25

### Association between participant characteristics and health facility delivery

A statistically significant association was found between educational status, wealth quintile and area of residence, and health facility delivery (*p* < 0.05). In addition, geographical region, religion, health insurance status, partner’s educational status and distance to health facility were associated with health facility delivery (*p* < 0.05). Exposure to mass media was associated with health facility delivery (*p* < 0.05). Young mothers in the Upper East Region had the highest prevalence of health facility delivery (91%). Home delivery was common among participants in the Northern Region (60%), those who professed Traditional religion (72%), and those who obtained zero ANC visit (84%) (Table 2).

**Table 2:** Cross-tabulation of socio-demographic characteristics and place of delivery

Characteristic	Total n	Home delivery n (%)	Facility delivery n (%)	Chi-square	*P*-value
**Age (years)**					
15–19	178	36(20)	142(80)	3.40	0.16
20–24	686	185(27)	501(73)		
**Educational status**					
No education	154	72(47)	82(53)	66.79	< 0.01
Primary	203	65(32)	138(68)		
Senior high	496	84(17)	412(83)		
Higher	11	0 (0)	11(100)		
**Marital status**					
Unmarried	320	67(21)	253(79)	5.51	0.06
Married	544	152(28)	392(72)		
**Wealth index**					
Poorest	187	86(46)	101(54)	98.81	< 0.01
Poorer	215	67(31)	148(69)		
Middle	221	55(25)	166(75)		
Richer	155	9(6)	146(94)		
Richest	86	3(4)	83(96)		
**Areas of residence**					
Urban	341	34(10)	307(90)	77.53	< 0.01
Rural	523	36(188)	335(64)		
**Region**					
Western	95	26(27)	69(73)	101.46	< 0.01
Central	102	31(30)	71(70)		
Greater Accra	110	13(12)	97(88)		
Volta	74	19(26)	55(74)		
Eastern	101	32(32)	69(68)		
Ashanti	129	14(11)	115(89)		
Brong-Ahafo	97	16(17)	81(83)		
Northern	97	58(60)	37(40)		
Upper East	38	3(9)	29(91)		
Upper West	23	7(27)	16(73)		
**Religion**					
Christianity	669	154(23)	515(77)	6.28	< 0.01
Islam	133	33(25)	100(75)		
Traditional	21	15(72)	6(28)		
No religion	41	17(41)	24(59)		
**Employment status**					
Unemployed	302	72(22)	230(78)	2.75	0.12
Employed	562	152(27)	410(73)		
**Covered by health insurance**					
No	326	101(31)	225(69)	9.70	< 0.01
Yes	538	118(22)	420(78)		
**Husband/partner education**					
No education	130	56(43)	74(57)	25.86	< 0.01
Primary	75	24(32)	51(68)		
Senior high	379	91(24)	288(76)		
Higher	26	2(7)	24(93)		
**Distance to a health facility**					
Big problem	250	83(33)	167(67)	11.08	< 0.01
Not a big problem	614	135(22)	479(78)		
**Frequency of reading newspaper**					
Not at all	774	209(27)	565(78)	14.27	< 0.01
Less than once a week	53	4(7)	49(93)		
At least once a week	35	5(13)	30(87)		
**Frequency of listening to a radio**					
Not at all	161	55(34)	106(66)	2.98	0.05
Less than once a week	301	75(25)	226(75)		
At least once a week	402	88(22)	314(78)		
**Frequency of watching television**					
Not at all	233	79(34)	154(66)	19.21	< 0.01
Less than once a week	234	63(27)	171(73)		
At least once a week	397	75(19)	322(81)		
**Antenatal care (ANC)**					
No visit	24	20(84)	4(16)	82.90	< 0.01
1–3 visits	121	52(43)	69(57)		
4–7 visits	520	120(23)	400(77)		
8–20 visits	200	28(14)	172(86)		

*P-value < 0.05

### Predictors of health facility delivery

Participants who had secondary school education had higher odds of giving birth in a health facility (AOR = 3.5, 95% CI: 1.33–9.23) compared to participants with no formal education. Participants residing in rural areas (AOR = 0.27; 95% CI: 0.14–0.514) had lesser odds of giving birth in a health facility compared with participants in urban areas. We also found that participants in the richest wealth quintile (AOR = 8.24; 95% CI: 0.95–71.77) had higher odds of giving birth in a health facility compared with those within the poorest wealth quintile. Also, participants who obtained zero ANC visits (AOR = 0.00; 95% CI: 0.00–0.01) had lesser odds of accessing skilled delivery services compared with those who obtained 8 or more ANC visits. Participants in the Upper East region (AOR = 8.44; 95% CI: 2.33–30.55) had higher odds of giving birth in a health facility compared with those in the Western region (Table 3).

**Table 3:** Binary logistic regression of predictors of health facility delivery among young women in Ghana

Covariate/exposure	Adjusted analysis OR (95% CI)	Adjusted Wald *p*-value
**Educational status**		
No formal education	1(reference category)	0.02
Primary	1.67 (0.72–3.89)	
Secondary	3.50 (1.33–9.23)	
**Wealth index**		
Poorest	1(reference category)	0.02
Poorer	1.83 (0.98–3.38)	
Middle	2.32 (0.94–5.75)	
Richer	9.64 (2.49–37.17)	
Richest	8.24 (0.95–71.77)	
**Areas of residence**		
Urban	1 (reference category)	< 0.01
Rural	0.27(0.14–0.514)	
**Region**		
Western	1(reference category)	< 0.01
Central	0.90 (0.31–2.63)	
Greater Accra	1.61 (0.35–7.34)	
Volta	1.14 (0.34–3.80)	
Eastern	1.11 (0.37–3.32)	
Ashanti	1.12 (0.50–9.00)	
Brong-Ahafo	2.57 (0.66–9.98)	
Northern	0.78 (0.22–2.79)	
Upper East	8.44 (2.33–30.55)	
Upper West	3.10 (0.82–11.68)	
**Religion**		
Christianity	1(reference category)	0.12
Islam	1.27 (0.64–2.49)	
Traditional	0.13 (0.02–0.96)	
No religion	0.76 (0.34–1.69)	
**Covered by health insurance**		
No	1(reference category)	0.17
Yes	1.45 (0.85–2.48)	
**Husband/partner education**		
No education	1 (reference category)	0.23
Primary	0.92 (0.41–2.05)	
Senior high	0.57 (0.29–1.11)	
Higher	1.42 (0.32–6.43)	
**Distance to a health facility**		
Big problem	1 (reference category)	0.30
Not a big problem	1.27 (0.81–1.98)	
**Frequency of reading newspaper**		
Not at all	1(reference category)	0.26
Less than once a week	6.03 (0.52–69.79)	
At least once a week	0.68 (0.18–2.53)	
**Frequency of listening to a radio**		
Not at all	1(reference category)	0.58
Less than once a week	0.81 (0.39–1.66)	
At least once a week	0.67 (0.32–0.43)	
**Frequency of watching television**		
Not at all	1(reference category)	0.02
Less than once a week	0.35 (0.16–0.77)	
At least once a week	0.41 (0.19–0.84)	
**Antenatal care**		
8–20 visits	1(reference category)	< 0.01
4–7 visits	0.53 (0.27–1.03)	
1–3 visits	0.31 (0.14–0.69)	
No visit	0.00 (0.00–0.01)	

## Discussion

The results showed that 75% of the participants gave birth in a health facility. The prevalence of health facility delivery in this study is higher than what previous studies found in Nigeria (33.7%) [23] and rural India (46%) [24]. The difference in findings can be attributed to the implementation of the Free Maternal Health Care Policy in Ghana. With this policy, pregnant women can enrol on the National Health Insurance Scheme (NHIS) free of charge. Subscribers of the NHIS can access maternal health services, including skilled delivery services, free of charge [25]. Further, persons below the age of 18 years are exempted from paying the NHIS premium. There is evidence to show that the implementation of the NHIS has led to an improvement in the utilization of maternal health services [26].

Notwithstanding, a considerable proportion (25%) of young mothers delivered at home. Home delivery predisposes the mother and the new born to a high risk of infections and death during and after delivery. Home delivery is also associated with late initiation of breastfeeding and inadequate postnatal checks for the mother and the child [27]. Although skilled delivery service is free in Ghana, young mothers may still face some financial constraints, such as transportation costs. Also, evidence shows that insured persons in Ghana make unapproved payments when accessing health services (including maternal and child care services) in NHIS accredited health facilities, which may pose as barriers to accessing skilled delivery services [28]. Moreover, adolescent mothers are at a higher risk of intrapartum mistreatment during childbirth [29]. These factors may explain why some young mothers deliver at home. This finding calls for urgent attention from stakeholders, including the Ministry of Health, Ghana Health Services, and Non-Governmental Organizations that are interested in promoting the health and wellbeing of women.

In addition, it was found that health facility delivery was associated with higher maternal education and higher socioeconomic status. Also, young mothers residing in rural areas had reduced odds of utilizing skilled delivery services. All the above findings have been supported by similar studies in India [24], Nigeria [23], and Bangladesh [30]. For instance, a systematic review of similar studies across low-and middle-income countries confirms that adolescents’ utilization of maternal health services is influenced by educational status, wealth index, and place of residence [31]. These findings are understandable since women with higher education are literates and can access information about pregnancy and childbirth complications from the electronic and print media. Moreover, women from wealthy households are more likely to afford health services compared with those from poor households. In Ghana, the majority of the health facilities are concentrated in urban areas, hence women in these areas are more likely to have access to skilled delivery services. Further, young mothers in the Upper East region had the highest odds of giving birth in a health facility. Available evidence shows that the Upper East region has the highest coverage of maternal health services, including antenatal and skilled delivery [17]. Further, this study demonstrated that utilization of skilled delivery was associated with adequate ANC visits. Nigerian adolescent mothers who accessed full antenatal care visits were seven times more likely to give birth in a facility compared with their counterparts [23]. There is substantial literature to show that ANC visits and skilled delivery are positively correlated [31].

The findings from this study have implications for maternal health policy and programming.

This study highlights the socio-economic and geographic disparities in the utilization of skilled delivery services among young mothers. Utilization of skilled delivery services was low among young mothers with low educational status, those from poorer households and those residing in rural areas. This implies that these young mothers are at a higher risk of pregnancy and childbirth complications as well as negative birth outcomes. It is, therefore, imperative for stakeholders, including the Ministry of Health and Ghana Health Services, to prioritize maternal health interventions among young women from low socio-economic backgrounds. Interventions that focus on increasing ANC coverage, promoting girl child education, and bridging the socio-economic gap between the rich and the poor will help increase the utilization of skilled delivery services among young women in Ghana. Stakeholders must ensure equity in the distribution of health care resources, including infrastructure and health workforce, between rural and urban areas.

### Strength and limitations

To the best of the authors’ knowledge, this is the maiden study in Ghana to assess the utilization of skilled delivery services among young mothers, using national representative data. Therefore, these findings can be generalized to the young population in Ghana. However, this study is not without limitations. This study employed a cross-sectional design, therefore, the findings cannot explain the reasons behind young mothers’ choice of place of delivery. Hence, studies that employ qualitative designs to explore in-depth the intricate views of adolescent and young mothers regarding the choice of place of delivery will be valuable. The associations found in this study, as with surveys in general, do not mean causation. Thus, the findings must be interpreted with caution.

## Conclusion

The findings of this study showed that the majority of young mothers in Ghana utilized skilled delivery services. Yet, a substantial proportion of young mothers delivered at home, despite efforts to increase the utilization of maternal health services. Health facility delivery was influenced by socio-demographic factors, socio-economic factors, and adequate utilization of ANC. Going forward, interventions that seek to increase the utilization of skilled delivery services among young women should focus on addressing the socio-economic disparities. Stakeholders can leverage these findings to help reduce maternal and neonatal mortalities as well as promote the health and wellbeing of young women in Ghana.

## Data Availability

The data used in this study is owned by The DHS Program, therefore, the authors cannot share the data. Interested persons can contact The DHS Program for the data (https://dhsprogram.com/data/available-datasets.cfm). The authors confirm they did not have any special access or privileges to the data that other researchers would not have.

## Abbreviations

GDHS: Ghana Demographic and Health Survey
ANC: Antenatal care
NHIS: National Health Insurance Scheme
SSA: Sub-Sahara Africa
MMR: Maternal Mortality Rate
FMHCP: Free Maternal Health Care Policy
